# School-Based Mental Health Initiative: Potentials and Challenges for Child and Adolescent Mental Health

**DOI:** 10.3389/fpsyt.2022.866323

**Published:** 2022-06-09

**Authors:** Kelly Y. C. Lai, Se-Fong Hung, Hannah W. S. Lee, Patrick W. L. Leung

**Affiliations:** ^1^Department of Psychiatry, The Chinese University of Hong Kong, Hong Kong, Hong Kong SAR, China; ^2^Department of Psychology, The Chinese University of Hong Kong, Hong Kong, Hong Kong SAR, China

**Keywords:** mental health, school-based services, schools, teachers, healthcare professionals

## Abstract

School-based mental health support services allow children and adolescents easy access to services without requirement of traveling to clinics and hospitals. We describe a School Mental Health Support Scheme (SMHSS) piloted in Hong Kong and discuss the challenges and learnings from the experience. This conceptual paper argues that accessibility is not the only advantage of such services. Teachers are significant others in child development, alongside with families. They play a central role in impacting the children's/adolescents' needs for competence and adult attachment, while schools provide an expanded social network of peers for one's social relationship. The fulfillment of these needs has powerful implications in the mental health of the children/adolescents. Teachers can help students to develop a sense of competence with self-worth and self-identity *via* providing guidance and feedback, whether they be on one's strengths or weaknesses, with acceptance, tolerance and unconditional positive regard. Particularly, the latter define a form of teacher-student relationship or adult attachment that offers the children/adolescents emotional security and nourishment, protecting them from failings and adversities. Teachers can also supervise and guide their students' social development with peers at schools. A recent meta-analysis has found preliminary evidence that those school-based mental health services integrated into the teachers' routine teaching activities are more effective. Teachers, who are overworked and stressed by the schools' overemphasis on academics and grades, have yet to fully grasp their unique roles in supporting students with mental health needs. This paper ends by advocating a paradigm shift in which both the healthcare professionals and educators should forge a mutually beneficial collaboration in jointly enhancing the mental health of children/adolescents at schools.

## Introduction

In our highly competitive world, children and adolescents are living in an increasingly stressful environment, with the need to succeed permeating down from the adult world to the world of adolescents and even very young children. Educational achievement is pushed to the forefront of children's lives, creating stress which hugely impacts their mental health, physical health, family relationship, and social relationship (1, 2). By adolescence, one in seven youths was diagnosed with at least one type of mental disorders (3, 4). Kessler et al. (5, 6) reported that most adult mental health disorders began during childhood and adolescence, with half of the adult cases having an onset at the age of 14 or before. A more recent review showed an even earlier onset (7). To make matters worse, only around 20% of these cases received treatment (8). Without effective treatment, adverse effects were found to persist into adulthood, (7) impeding growth, life opportunities, and wellbeing (5). As a result, effective preventative measures and prompt intervention during childhood and adolescence should be the top priorities in the delivery of mental health care.

Besides families, schools represent the second social environment where children and adolescents spend a lot of their time, and on which a large part of their learning and development depends. Given the above, schools become the venue where early signs of mental health problems can be detected and where access to intervention can be provided promptly (9). This recognition has led to the rise of school-based mental health services, with the aims of promoting mental health awareness, as well as prevention and amelioration of students' mental health problems (10). A recent meta-analysis reported preliminary supportive findings of small-to-medium effect sizes of school-based mental health services in reducing student mental health problems, but strategies that were specifically integrated into teachers' routine teaching activities recorded a larger effect size (11). Yet, teachers were often uncertain about their roles in supporting their students with mental health problems (12). Some even felt that it was beyond their roles since they were not trained in mental health (13).

This present conceptual paper will discuss critically the roles of schools and teachers in the development of children and adolescents. We will review one pilot School Mental Health Support Scheme (SMHSS) in Hong Kong as an example to highlight the challenges and learnings, and look to the ways forward. We will advance the thesis that ready access to mental health services for children/adolescents at schools is not the only advantage of school-based mental health services. Teachers at schools have powerful and unique roles in impacting child development, including mental health, which cannot be substituted by mental health professionals at hospitals/clinics. Above all, a paradigm shift is suggested, in which healthcare professionals and school administrators/teachers should take on an expanded vision of their roles and functions and make a coordinated and mutually beneficial effort to support and optimize the development of students with mental health problems at schools.

## Background Of The Student Mental Health Support Scheme (Smhss) Piloted In Hong Kong

The Student Mental Health Support Scheme (SMHSS) is a pilot school-based program conducted in Hong Kong mainstream schools to examine its feasibility, reactions from different stakeholders, and potential challenges and obstacles. In the following, we will first discuss academic performance as one key stressor for children and adolescents, particularly in Hong Kong, to be followed by a succinct description of the pilot SMHSS, and finally its main challenges and learnings.

### Education in Danger of Becoming a Single-Minded Pursuit on Academic Excellence

Currently, there is a growing trend worldwide for schools to put greater sole emphasis on academic performance. The rise of such a trend is urged on by the vigorous competition among students since good academic achievement means entry to better universities, improved job prospects, and improved financial stability (14). These concerns lead to a similarly rising trend of competitions among schools regionally and nationally since academic scores are often taken to measure the success of schools (15). These high-stake assessments and competitions place a massive amount of stress on schools, which in turn push students hard to perform well academically (16, 17).

When education becomes a single-minded pursuit of academic excellence, it can “make or break” a child. Students who are less academically able may feel side-lined, demoralized, and inferior (18). They may face frequent criticisms from adults (19) or bullying and discrimination from their peers (19–22). In the drive to pursue academic excellence, students' non-academic potentials may be overlooked and alternative opportunities to foster their development neglected. Growing up under such relentless pressures to achieve, some students inevitably develop mental health problems, which, in turn, can lead to academic under-performance, thus running a downward spiral (23, 24).

In Hong Kong, the pursuit of academic excellence is particularly prominent with wide ranging ramifications, given that such value on academics is rooted deeply in traditional Chinese culture. It is also seen as evidence of well-functioning children and successful parents (25, 26). Children rendering poor academic performance are often frowned upon, blamed for their lack of efforts, and labeled as a “failure,” leading to a sense of guilt in themselves and a loss of “face” for their families. These views lead to a strong goal orientation and intense competition among Hong Kong students, whose purpose of learning is to out-perform others instead of enjoying intrinsically the learning processes (27).

As a result, immense pressure is piled onto Hong Kong children from a young age. At schools, children face regular tests and examinations. With endless amounts of homework, drilling, and extra tuition, Hong Kong students have little time for relaxation and leisure activities (28). Academic goals become their sources of high anxiety (29, 30). A 6-year longitudinal study particularly indicated that Hong Kong adolescents suffered from increasing hopelessness and reduced life satisfaction (31).

Yet, over-emphasis in academic excellence and grades is not a phenomenon specific to Hong Kong. It is also prevalent in eastern and southern Asian countries, like Singapore, (32) South Korea, (28) or even in Western countries like the USA (33). This overemphasis not only creates stress for students, but also equal stress on teachers, who thus devote a large amount of classroom time to deliver academics and prepare students for tests and examination (34). This in turn results in depriving students of other social experiences and interactions (35). Most teachers do care for the holistic development of children, but they also feel that they already have too many responsibilities, are overworked, and highly stressed (36).

### Mental Health Demands and Services for Children and Adolescents in Hong Kong

In Hong Kong, mental health services rely heavily on specialist Child and Adolescent Mental Health Services (CAMHS), which are publicly-funded and hospital-based. While there are community services providing mental health care, these tend to be piecemeal, lacking proper organization and support to them. There is also a lack of collaboration among hospital-based specialists and community practitioners, as well as across different professional disciplines involved in the care of children and adolescents, such as healthcare, education, and welfare. This lack of coordination means that the efforts and effectiveness of mental health care for children and adolescents are severely hampered. Yet, schools represent a social environment where significant challenges and opportunities on child development pervade and therefore presents an appropriate setting for coordinated intervention to take place. Furthermore, it is becoming increasingly evident that the single-minded focus on academic pursuit has serious mental health consequences for Hong Kong students. Against this background, a pilot school-based mental health support service, SMHSS, is developed to strengthen the support to students with mental health problems and to maximize the schools' potentials to positively influence the development of their students. Mental health problems, within the remit of the SMHSS, are broadly defined and include psychiatric symptoms of varying severity, ranging from subclinical to full-blown clinical levels. The full range of psychiatric disorders such as attention-deficit/hyperactivity disorder, autism spectrum disorder, oppositional defiant disorder, conduct disorder, anxiety disorders, depression and psychosis are included.

### Aims and Operation of the SMHSS

With an emphasis on multi-disciplinary collaboration within the school setting, the SMHSS aims at (1) strengthening the support to students with mental health problems through early identification and prompt access to interventions, (2) equipping teachers and school social workers with the knowledge and skills to provide support to these students, and (3) encouraging and empowering schools to provide a more nurturing and less stressful environment for all students. In each participating school, a pledge of full support must be committed by the school senior management (Principal/Vice-Principal). A multi-disciplinary team is set up to coordinate services at schools, with an Advanced Practice Nurse (APN) from the public-hospital CAMHS, a designated teacher [i.e., special educational needs coordinator (SENCO)], and a school social worker as core members. SENCOs in Hong Kong are teachers who are given additional training and protected time to cater for the special educational needs (SEN) of students. These students are those with physical and mental disabilities, including mental disorders such as anxiety, depression, attention-deficit/hyperactivity disorder, autism spectrum disorder, etc. Besides the SENCO, other involved school personnel, e.g., other teachers or the school social worker, do not have protected time. The schools also do not have extra funding. The multi-disciplinary team is responsible for assessing and managing the mental health, educational and psychosocial needs of students with mental health problems, and provides the indicated treatment and school-based support. This team also coordinates required services to be provided by other school teachers and educational psychologists, as well as specialists of hospital-based CAMHS, including psychiatrists and clinical psychologists. These hospital-based specialists are encouraged to communicate directly with the school personnel for updates and discussions to forge a close working relationship with the school personnel. The students' progress is monitored through regular multi-disciplinary case conferences held 3–4 times during the school year, as well as working meetings as and when required.

The role of different professionals is delineated as much as possible to reduce duplication and maximize expertise. The SENCOs, with the advice and support of educational psychologists, work with other teachers to implement educational-based management plans, and monitor students' progress. Students' educational needs may be supported by, for example, special remedial measures, as well as strategies to boost confidence and self-esteem, reduce study stress, and improve motivation. School social workers provide socio-emotional support to the students and their parents. They also help link up community resources to match the students' and their families' needs. When necessary, family intervention is referred to relevant outside social service agencies.

Training is provided to participating schools to enhance their knowledge on student mental health and relevant management skills through a 30-h formal series of lectures, workshops and hospital-based clinical attachments. Informal channels of training are effected through working meetings, multi-disciplinary conferences, *ad hoc* discussions and experience sharing, which help to put knowledge into practice, and fine-tune assessment and intervention skills at schools.

The SMHSS is spearheaded and coordinated at the highest level by the government ministry on health care in Hong Kong, namely, the Food and Health Bureau. It establishes a coordinating task force involving relevant disciplines from various government ministries (e.g., the Education Bureau and the Social Welfare Department), statutory bodies (e.g., Hospital Authority), and non-governmental organizations (e.g. NGO), etc.

### School and Student Recruitment

Invitation was extended to all schools in Hong Kong by the Education Bureau to solicit their interest in participating in the pilot phase of the SMHSS. Interested schools were then selected by the task force, taking into account a range of factors such as each school's estimated number of students requiring the services of the SMHSS, and the workload that could be managed by the SMHSS teams. A balance was also sought to select schools from across the whole of Hong Kong so as to reduce the bias toward particular socio-economic strata.

Students supported by the SMHSS are identified through two routes. One is through nomination by teachers and school social workers. Another route is through a multi-tiered screening program using Spence Children's Anxiety Scale (SCAS) (37) to identify students with anxiety symptoms. The SMHSS protocol recommends screening of students in the fourth year of primary school (aged 9–10) and the first year of the secondary school (age 12–13), but schools can opt to screen another student year if they wish.

## Challenges And Learnings From The Pilot Run Of The Smhss

Most published studies on school-based mental health services are from Western countries. Their application in non-Western societies is still relatively understudied, with the exception of one recent Singapore study (38). Cultural differences, such as expectations of children's behavior, the emphasis on academic achievement, and the stigma of mental illnesses in different societies, can influence how mental health services are perceived and received at schools. In the following sections, we will discuss the challenges experienced and learnings accumulated from the delivery of the SMHSS in Hong Kong, and suggest ways forward.

### Feedback From Stakeholders

Teachers and school social workers appreciate the support provided by healthcare professionals in advising and managing the needs of the students with mental health problems. They find access to clinical information easier and have developed a better understanding of the students' needs. Students and parents in return feel a more supportive school environment. Some students, however, express reservations about skipping lessons to attend the intervention sessions conducted at schools.

Healthcare professionals find working within schools challenging because of the need to understand the culture of the collaborating schools, the interpersonal dynamics, and the personalities involved in order to build up trust that renders the collaboration effective. This requires repeated dialogues, exchanges and sharing. With time, they feel that schools are able to develop a more holistic understanding of the impacts of mental health symptoms on students' functioning and be more accommodating and supportive. Working within the schools also helps them contextualize the students' difficulties and find ways of support that are feasible and relevant, fitting in with what the schools and the families can provide or accept (10). Understanding both the individual and environmental contexts makes dialogue between healthcare professionals and school personnel more relevant and meaningful. In contrast, working solely within hospital-based services, such as the specialist CAMHS, the healthcare professionals may not really know about the real life of the children and adolescents as students outside the hospital setting and thus hamper the applicability of their intervention.

Yet, healthcare professionals are struck by some schools' view that it is the remit of healthcare professionals to provide intervention. Furthermore, arranging a time to meet with the students can also be difficult, dependent on the students' schedules, willingness to skip lessons, and teachers' willingness to release them from lessons. These logistic difficulties mean that healthcare professionals cannot readily meet the students as expected, despite that they are physically present at schools. All these reflect the tussle of priorities between mental health and academics. It is a mindset that needs to be addressed since academic achievement and mental wellbeing are not mutually exclusive choices to be made, but are themselves highly interlinked.

### Case Nomination, Screening, and Workload

Nomination by teachers and school social workers is one of the two routes students with mental health problems are referred to the SMHSS. The clinical profiles of the nominated students confirm that they have significant psychopathology, with the majority suffering from neurodevelopmental, anxiety and depressive disorders. Schools are mostly concerned about their emotional and behavioral problems, school attendance problems, study stress, and self-harm behavior. Our experience echoes that of existing literature that teachers and school social workers are well-placed to identify students with mental health needs.

Most of the students identified through screening turn out to have clinically significant anxiety symptoms, but they had not consulted mental health professionals in the past, and were not suspected to have mental health problems by their schools. Further assessment finds most of them to be in need of intervention. However, the percentages agreeing to receive intervention were low, below 60%. Various barriers to services, e.g., stigma, probably exist. The issue of stigma will be discussed in a later section.

Screening brings up many workload and logistics issues. From the schools' perspective, issues such as scheduling the screening exercises into the busy school calendar, allocating time and staff to supervise the questionnaire completion, and the subsequent follow-up of these students are perceived to be burdensome. Crucially, screening uncovers many students previously not suspected to have mental health problems and schools do not feel they have the resources to cope with the extra caseloads. These problems have dampened the enthusiasm for screening. However, screening allows early identification and early intervention. It is essential that school administrators are aware of its benefits, so that adequate manpower and resources can be found to ensure that schools have the capacity to provide more dedicated support to the students who have hitherto undiagnosed mental health problems. However, this is easier said than done. In the sections below, we will return to these school resource and capacity issues in greater depth.

### The Challenge of a Paradigm Shift for Healthcare Professionals and School Administrators/Teachers

#### The Roles of Mental Health Professionals in the SMHSS

Our experience of this pilot trial finds that it is still the healthcare professionals who are primarily involved in delivering intervention and support, while the direct involvement of school personnel is still less than expected. The aim of the SMHSS to empower and build up schools' capacity to support their students is not gaining a great deal of mileage. It may be that old habits die hard and do not get changed easily. There is a tendency for the healthcare professionals to become too “enthusiastic” and take up too much work at schools. This unwittingly goes against one main aim of the SMHSS to build schools' capacity *via* ample participation of the school personnel in the Scheme. Working toward a paradigm shift, a more precise collaborative framework with further well defined roles should be drawn up. Mental health professionals need to remember that the needs of children and adolescents with mental health problems are complex, and are often linked to different contexts and systems. Keeping a focus on empowerment and effective collaboration, healthcare professionals need to have multiple perspectives of their role. First, in fitting with their professional training, they assess and diagnose the mental state and the areas of concern of the referred students. Second, they contextualize the students' needs and difficulties within the students' family and school systems, therefore introducing an ecological perspective and bringing into focus the interactive nature of those risk factors in impacting the students' functioning. This emphasis on the influences of different systems helps put the need for multi-disciplinary collaboration into context. Third, by increasing the schools' knowledge and understanding of mental health problems and how they impact on students' functioning, and through the active involvement of school personnel in supporting their students, they encourage schools to pay attention to their student's mental health and to be cognizant of the benefits of a supportive and accepting school environment on students' functioning. All these perspectives need to be borne in mind so as to put in focus the need for collaboration and the complementary roles of healthcare, educational, and social care professionals in impacting and optimizing students' development, including mental health. The results of a recent meta-analysis of the effectiveness of school-based mental health services echo similar implications (11). Direct involvement of healthcare professionals in running the services at schools does not necessarily produce the best results. Instead, mental health services integrated into teachers' regular teaching activities produce a larger effect size.

#### The Roles of Teachers/Schools on Child Development

Teachers, on the other hand, need to truly recognize the important roles they play in their students' development. Schools are charged with the responsibility of providing an environment for children to learn, where they develop their potentials, recognize their strengths and weaknesses, build self-confidence and a sense of mastery, and cultivate a life orientation. It is a place where they build up resilience by developing skills to manage life's many challenges. These are the developmental tasks that are crucial during childhood and adolescence. Erik Erikson (39, 40) proposed two psychosocial challenges during these periods: industry vs. inferiority in childhood, and identity vs. role confusion in adolescence. Successful coping to these two challenges results in a sense of industry in which children are confident of their abilities and are willing to challenge themselves, as well as a sense of identity that includes an awareness and acceptance of one's strengths and weaknesses. Ryan and Deci (41, 42), in their self-determination theory, supplemented Erikson's theory by highlighting three basic psychological needs, namely, the need for autonomy, competence, and relatedness. Schooling necessarily puts students in a situation, where their performance is evaluated and compared with peers by teachers. In consequence, the students' sense of competence is determined not only by their own actual task accomplishment, but also by teachers' or schools' expectations and feedback (43). Thus, teachers' or schools' verdicts of academic success result in a sense of industry and confidence about their abilities. Contrarily, when the verdicts are negative, students feel inferior, thus undermining the development of their self-esteem and sense of competence. This is where teachers or schools, as inevitable performance evaluators, have their unique and powerful roles in impacting students toward or away from meeting their developmental challenges. The pursuit of academic grades may be a worldwide reality, yet in providing feedback, whether it be on one's strengths or weaknesses, teachers can show their acceptance and unconditional positive regard toward their students (44, 45). It is to accept the students as who they are, regardless of their ability, achievements, background, and character (46). When teachers do so, it separates the students' behavior and performance from their innate, original self, allowing them to feel inherently valued and respected regardless of their mistakes, flaws, or performance. Furthermore, teachers' acceptance allows students to feel secure, which in turn encourages them to explore their limits and discover their abilities without fear of judgement or rejection (47). They can be bolder to pursue their full potentials and become resilient toward future challenges (47). By acting as nurturing and significant adults, teachers encourage students to develop a trusting relationship with them. Such secure attachment and meaningful relatedness will foster students' positive development, including mental health.

Teachers as attachment figures are especially important for at-risk students, when their parents or families are unable to provide the secure attachment, or who have experienced adversities in growing up (48). Under these circumstances, teachers can act as alternative, substitute attachment figures to provide the security and trust, which can buffer against the damages resulting from behavioral, psychosocial, and demographic risks, such as poverty, childhood abuse, maternal depression, as well as restore behavioral, psychological and social functioning (49–51). At-risk children and adolescents can learn that despite their adverse family environment, yet in another social environment, i.e., schools, they can be cared for and nurtured by their teachers, laying a foundation for healthier development.

Rutter et al. (52) published a landmark study, way ahead of its time, on the significant impacts of schools on the behavior and attainment of their students. Unfortunately, there has not been a huge literature ensuing. Yet, from those available studies, they are generally supportive of the importance of schools/teachers in facilitating or impeding students' development. For example, teachers demonstrating high levels of acceptance, warmth, and unconditional positive regard had students who showed positive educational outcomes, self-competence, motivation, and achievement, (53) as well as psychological adjustment, (54) even among those from disadvantaged background (52). Rucinski et al. (55) reported lower rates of depressive symptoms and externalizing behaviors such as aggression when the quality of teacher-student relationship was positive. The latter also helped to free students from discrimination and stigmas, and increased their help-seeking behavior (56).

Indeed, teachers may be well aware of the impacts mental health problems have on their students' functioning, and the support these students need. Unfortunately, our local teachers are often overworked with large classes (up to 40 students per class), and are both physically and mentally stressed out by fierce competition in securing high grades for their students, the attainment of which defines their success and career as teachers. This narrows the teachers' focus of themselves as mere imparters of book knowledge. Being highly stressed, they are unlikely to exhibit an “enthusiasm” to broaden their roles to be educators of the whole person, or to manage their students' difficulties arising from their mental health problems. These stress, predicament, and ambivalence of our teachers are not likely to be unique to Hong Kong. To address these issues, educators, including school administrators and teachers, may need to revisit and re-kindle their broader vision as educators. This is very much in line with recent calls in other developed countries like the UK and Australia for an educational reform or a policy change in which both academic achievement and students' wellbeing should be focused (28, 57).

With a broad vision in education, a multi-tiered system of mental health care within schools can be established to span prevention to targeted intervention (58). At the universal preventive level, school administrators/teachers, school social workers, as well as students can be mobilized to help develop school-wide strategies to build a nurturing, accepting and tolerant school climate so that students can feel safe, connected, supported, and involved (59, 60). This would be in keeping with Ryan and Deci's theory of the need for relatedness for optimal psychosocial development of each individual (41, 42). At a program level, teachers are well-placed to help design and deliver mental health awareness and promotion programs, as well as to incorporate into their regular curriculum activities to build students' resilience by teaching emotion regulation, problem-solving and social skills, (17) as well as providing the opportunities for students to develop their talents in non-academic areas. Government education officials and school administrators must be fully cognizant and supportive of such needs in the school curriculum. Within the classrooms, teacher-administered behavioral management and psychosocial intervention have been shown to be effective in improving students' externalizing and internalizing behaviors (61). Involving teachers in the delivery of these programs can empower them to have a more prominent role in supporting students, and promote a school and classroom environment that is accepting, nurturing, and conducive to positive learning experiences and good mental health. Furthermore, evidence-based treatment for specific disorders such as anxiety and depression can be delivered in group or individual format by trained school social workers or trained teachers. Training can be provided by mental health professionals, whose roles can be more consultative, assisting in case conceptualization, providing advice, training, and supervision on intervention, and hence building schools' capacity. When clinical needs arise, they will still be on hand to provide direct intervention and hospital-based services. Adopting this multi-tiered approach will enable each discipline, including mental health, education and social work, to better delineate their roles and to contribute meaningfully and effectively within their areas of professional expertise and spheres of influences in a coordinated manner (62).

#### The Challenge of Stigma and Priorities

Stigma is one of the critical barriers to help-seeking (63). The dissonance between one's preferred self/social identity and the stereotypes of mental illnesses (e.g., mental illnesses representing personality weakness, being “not normal,” or not trying hard enough) leads individuals to anticipate negative consequences such as shame, rejection, and discrimination. Worries about disclosure and confidentiality also contribute to the reluctance to seek help (63).

Furthermore, the Chinese culture is regarded as a “face” culture, in which “face” represents one's public image and reputation, so that one's worth is bestowed by others. Compared to western cultures, Chinese culture endorses a higher level of stigma toward mental illnesses, and this difference is accounted for by the concern about “face” (64). Chinese parents of children with mental illnesses are prone to self-stigma as they perceive a lowering of their social standing (65). They are also quick to internalize stigma with a sense of self-blame and responsibility (66). Children brought up in this “face” culture can develop self-stigma from a young age, (67) which is generated from the public stigma that they experience in the community.

The concern about stigma and its influence on help-seeking are evident among students supported by the SMHSS. Some indicate a reluctance to meet healthcare professionals during the school days, as they are worried that other students may notice and start gossiping. Some of the students screened positive will outright decline intervention because of such concern. These findings echo those identified in the western literature (68, 69). Therefore, although providing services in schools allows easier access, the proximity of peers and teachers, and the worries about confidentiality and negative labeling actually work against the willingness to receive services.

In Hong Kong, given the high priority given to academic excellence, another reason often provided for declining intervention is the need to keep up with academic demands. Some students express that they do not want to skip lessons to meet healthcare professionals, while some teachers do not want to release students during lessons. This tussle of priorities between academics and mental health, as if they are mutually exclusive and there has to be a choice one over the other, have to be addressed head on. The SMHSS should make enhanced efforts to educate about mental illnesses and reduce stigma. School administrators/teachers, parents, and students should be guided to understand the reciprocal negative influences of mental illnesses and academic functioning over each other. Such knowledge may prompt them to accord more urgency to improving mental health and be more willing to accept or render help, while setting realistic and achievable academic goals.

The environment in which help is provided is also important. Adolescents are more likely to seek help if they feel able to trust those from whom they seek help, and this trust includes ensuring confidentiality, feeling listened to and taken seriously, while the care-providers are caring and non-judgmental (69). If the teachers can provide such trusting and accepting environment at schools for those students in need of mental health services, the concerns for stigma and the artificial competing priorities between academics and mental health can be much allayed. The students can feel less hesitant about help seeking and reap the benefits of a more accessible SMHSS.

#### The Challenge of Manpower and Sustainability

The model of the SMHSS is manpower intensive. It creates extra workload for both teachers and school social workers, when they are expected to become more involved in providing support for students who are already known to have mental health problems. The screening at schools also uncovers more students who are previously not known to have mental health problems. The SMHSS also generates extra workload for hospital-based CAMHS because of the requirement to provide clinical training/supervision at schools. Given the unavoidable limited manpower available, its long-term sustainability needs to be considered. First of all, the number of students directly supported by the SMHSS has to be kept at a manageable level without compromising the quality and intensity of the intervention delivered. To achieve this, it is all the more important that the multi-disciplinary professionals involved must take on a paradigm shift to expand the vision of their roles. Healthcare professionals, for example, must see that there are significant others, e.g., teachers, whose involvement within their areas of expertise needs to be mobilized and empowered to support the varied and complex needs of children and adolescents with mental health problems. They have to reach out to them and take lead to forge a coordinated effort to support them. Teachers have the advantage of having a lot of contact time with their students and are therefore in a position to develop nurturing and accepting relationships with them, while at the same time, coaching them to gain a sense of mastery and self-confidence from the schooling experience, regardless of the presence or absence of mental health problems. These opportunities are unique to teachers at schools and cannot be replaced by healthcare professionals at hospitals. Therefore, the advantage of the SMHSS is not solely related to easy accessibility, and it is not a replacement for specialist CAMHS. More importantly, the SMHSS is intended to capitalize on the powerful influences of teachers/schools in optimizing students' development. The educators, school administrators and teachers alike, should thus take on a paradigm shift in expanding their roles, or in fact, resuming their broader vision, to acknowledge their potentials in being the significant others to impact their students' development. Furthermore, the sustainability of the model requires a political will from the senior government officials in health care and education at the highest level to acknowledge not only the important roles schools can play, but also to mobilize extra manpower and resources, e.g., protected time and extra funding, for the schools/teachers to proactively involve in the support of students' mental health needs. Despite the intensity of the manpower and resources required, healthcare professionals and educators delivering mental health services collaboratively at schools can generate a synergistic effect that benefits their students' development in multiple interrelated pathways: in education, competence, self-identity, mental health, and social relationship, etc, just to name a few. The outcomes will prove to be worthy of the added investment of resources to healthcare services and schools. Indeed, preliminary studies have already confirmed that teachers have a significant role in influencing their students' functioning and mental health (11). In future, health economics research needs to be conducted to indicate that the added resources for early assessment and intervention of students with mental health problems will bring considerable saving for the community in reducing the chronicity of their mental health problems and thus health cost, welfare cost, and loss of productivity, etc (38).

So far, the lessons from the pilot SMHSS in Hong Kong teach us that a paradigm shift from both our healthcare professionals and school personnel is vital for its success and long-term sustainability, as echoed in many other countries (12, 13). This shift is not entirely easy for a range of reasons, including old habits, unclear role delineation, heavy workload or priority tussle. It is going to take some time. More theoretical development, more supportive research, and more actual field trials may be required in order to get everyone on board with a paradigm shift toward a co-sharing/co-ownership, multi-disciplinary collaboration among educators and hospital-based healthcare professionals at schools.

## Conclusion

Mental health problems in children and adolescents have been posing a serious concern because of their high prevalence, complexities, and increasing service demand. In Hong Kong, a School Mental Health Support Scheme (SMHSS) was piloted with hospital-based healthcare professionals joining hands with school personnel to conduct mental health services at schools. Theories in child development have long suggested that besides family members, teachers are also significant others who can shape the developmental trajectory of children and adolescents. A school environment that provides accepting and nurturing relationships can foster their students' sense of competence, self-esteem and self-worth. Yet, many teachers/schools do not seem to leverage to the full their potential for positive impacts. As such, school-based mental health programmes should not only make services more readily accessible to children/adolescents. They should aim at drawing on teachers'/schools' powerful and unique roles in providing a school environment to meet the developmental needs of children and adolescents in competence, self-identity, attachment and peer relationship. The success in dealing with these developmental challenges is crucial for their mental health. These are roles and opportunities which cannot be taken over by healthcare professionals at hospitals. This conceptual paper draws on the lessons learned from running the SMHSS to call for a paradigm shift amongst hospital-based healthcare professionals and educators to broaden their vision and expand their roles to join force together at schools, capitalizing on their enormous potential in dealing with the sources where children's developmental needs may be unattended or frustrated with mental health problems emerging and perpetuating.

## Author Contributions

KL, S-FH, and PL conceptualized the themes and messages of this manuscript. KL and HL conducted the literature search and review as well as drafted the initial few versions. KL, S-FH, and PL finalized the manuscript, all involved in final editing and approval of the manuscript. All authors contributed to the article and approved the submitted version.

## Conflict of Interest

The authors declare that the research was conducted in the absence of any commercial or financial relationships that could be construed as a potential conflict of interest.

## Publisher's Note

All claims expressed in this article are solely those of the authors and do not necessarily represent those of their affiliated organizations, or those of the publisher, the editors and the reviewers. Any product that may be evaluated in this article, or claim that may be made by its manufacturer, is not guaranteed or endorsed by the publisher.
